# Episodic memory network characteristics in patients with amnestic mild cognitive impairment accompanied by executive function impairment

**DOI:** 10.1002/brb3.3601

**Published:** 2024-06-19

**Authors:** Chao Wang, Rukun Cheng, Wenhao Yang, Lin Qiu, Haifeng Liu, the Alzheimer's Disease Neuroimaging Initiative

**Affiliations:** ^1^ Department of Radiology Tianjin Baodi Hospital, Baodi Clinical College of Tianjin Medical University Tianjin China; ^2^ Department of Cardiology The Third Central Hospital of Tianjin Tianjin China; ^3^ Department of Radiology Tianjing Gong An Hospital Tianjin China; ^4^ Department of Radiology Liyang People's Hospital Changzhou China

**Keywords:** amnestic mild cognitive impairment, episodic memory network, executive function, episodic memory, functional connectivity

## Abstract

**Objective:**

To explore the functional connectivity (FC) characteristics of the episodic memory network (EMN) in amnestic mild cognitive impairment (aMCI) patients with different levels of executive function (EF).

**Methods:**

This study included 76 participants from the Alzheimer's Disease Neuroimaging Initiative database, comprising 23 healthy controls (HCs) and 53 aMCI patients. Based on EF levels, aMCI patients were categorized into aMCI‐highEF and aMCI‐lowEF groups. Cognitive function scores, pathological markers (cerebrospinal fluid β‐amyloid, total tau protein, phosphorylated tau protein, AV45‐PET, and FDG‐PET), and functional magnetic resonance imaging were collected and compared among the three groups. Seed‐based FC analysis was used to examine differences in the EMN among the groups, and partial correlation analysis was employed to investigate the relationship between changes in FC and cognitive function scores as well as pathological markers.

**Results:**

Compared to the aMCI‐highEF group, the aMCI‐lowEF group exhibited more severe cognitive impairment, decreased cerebral glucose metabolism, and elevated AV45 levels. Significant FC differences in the left superior temporal gyrus (STG) of the EMN were observed among the three groups. Post hoc analysis revealed that the aMCI‐lowEF group had increased FC in the left STG compared to the HCs and aMCI‐highEF groups, with statistically significant differences. Correlation analysis showed a significant negative correlation between the differences in FC in the left STG of aMCI‐highEF and aMCI‐lowEF groups and Rey Auditory Verbal Learning Test forgetting scores. Receiver operator characteristic curve analysis indicated an area under the curve of 0.741 for distinguishing between aMCI‐highEF and aMCI‐lowEF groups based on FC of left STG, with a sensitivity of 0.808 and a specificity of 0.667.

**Conclusion:**

aMCI‐lowEF exhibits characteristic changes in FC within the EMN, providing theoretical support for the role of EF in mediating EMN alterations and, consequently, impacting episodic memory function.

## Graphical abstract

The study found that amnestic mild cognitive impairment (aMCI) patients with lower executive function (aMCI‐lowEF) exhibit more severe cognitive impairment, decreased cerebral glucose metabolism, and elevated AV45 levels compared to those with higher executive function (aMCI‐highEF). Significant increased functional connectivity in the left superior temporal gyrus of the episodic memory network (EMN) were observed in aMCI‐lowEF group. These findings underscore the impact of executive function on EMN alterations in aMCI patients.

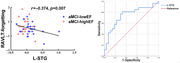

## 1 INTRODUCTION

Amnestic mild cognitive impairment (aMCI), which serves as a prodromal stage of Alzheimer's disease (AD), is considered an intermediate stage between normal aging and AD (W. Bai et al., 2022; Petersen et al., 2014). The core characteristic of aMCI is a decline in episodic memory (EM) function, closely associated with disease progression (Cova et al., 2017; Grande et al., 2021; Yue et al., 2023). Statistics indicate that approximately 10%–15% of aMCI patients convert to AD each year (Jessen et al., 2014). Timely detection and early intervention are crucial measures to delay the progression of aMCI to AD.

Increasingly, research reports non‐amnestic changes in aMCI, especially executive function (EF) (García‐García‐Patino et al., 2020; Harrington et al., 2013). EF refers to the ability to plan, organize, implement working memory, and switch between tasks. Harrington et al. (2013) proposed that alterations in cerebrospinal fluid β‐amyloid precede memory impairment, leading to a decline in EF. Several studies have found varying degrees of EF impairment in aMCI patients (Chow et al., 2022; Gagliardi & Vannini, 2022; Xu et al., 2020). Previous studies suggested that aMCI patients with EF deficiency constituted the high‐risk group for AD conversion (Tabert et al., 2006). Cerbone et al. (2022) found that participants with lower EF composite scores and greater EM severity at baseline predicted faster decline on dementia severity measures. Jung et al. (2020) have indicated that executive dysfunction in the frontal lobe is an adverse prognostic indicator for aMCI. aMCI with executive dysfunction in the frontal lobe exhibits more severe baseline cortical atrophy, suggesting a correlation between the presence of frontal executive dysfunction and more severe neurodegeneration. Van Dam et al. (2013) proposed that functional deficits in the anterior cingulate cortex (ACC) lead to a decline in EF and may potentially signify progression to AD. Yuan et al. (2016) highlighted EF as a key predictor for aMCI to AD conversion, with impaired EF exacerbating EM defects and increasing the risk of progression to AD. Ye et al. (2021) suggested that the EM defects in aMCI may be partially due to impaired EF. However, the mechanism by which EF in aMCI patients affects EM is not yet clear.

Resting‐state functional magnetic resonance imaging (rs‐fMRI) can reflect the intrinsic functional connectivity (FC) within brain regions and has been widely used in the study of the AD spectrum (Xue et al., 2021; Zhong et al., 2022). While early neuropsychological research on EM focused on the medial temporal lobe, particularly the crucial role of the hippocampus, the emergence of whole‐brain functional imaging has revealed that the EM process relies on highly interconnected neural circuits involving multiple brain networks and regions (Jeong et al., 2015; Moscovitch et al., 2016). This interconnected system is referred to as the EM network (EMN) (Liang et al., 2022; Shi et al., 2023). Multiple studies have previously found varying degrees of EMN impairment in the AD spectrum, which lead to cognitive decline (Cai et al., 2017; Cassady et al., 2021; Liang et al., 2022). Moreover, Ye et al. (2021) found a correlation between EF and EMN (F. Bai et al., 2016). However, whether EF influences EMN, thereby mediating EM function, remains to be investigated.

Therefore, according to EF, aMCI patients were divided into aMCI‐highEF and aMCI‐lowEF groups. The aims of this current study are to investigate changes in FC of EMN between three groups from three aspects: clinical manifestation, pathophysiology, and neuroimaging. The relationship between changes in the FC of the EMN and cognitive function was further studied. We hypothesized that there are different altered FC of EMN between aMCI‐highEF and aMCI‐lowEF groups, and changes of the aMCI‐lowEF group exhibit more significant changes resembling the pathological patterns observed in AD.

## 2 MATERIALS AND METHODS

### 2.1 Participants

The research data used for our study were sourced from the public database of Alzheimer's Disease Neuroimaging Initiative (ADNI) (http://adni.loni.usc.edu). In our investigation, a total of 146 patients with aMCI at baseline were enrolled. First, the EF composite score for 146 aMCI patients was calculated using the EF calculation model provided on the ADNI website. This model comprises tests including categorical fluency, trail‐making test parts A and B, digit symbol substitution test, digit span backward, and clock drawing (Gupta et al., 2020). In the present study, the mean of EF scores was 0.48, with a standard deviation (SD) of 0.95. Participants with a score higher than one SD above the mean EF score were classified as aMCI‐highEF, while those with a score lower than one SD below the mean EF score were classified as aMCI‐lowEF (W. Liu et al., 2021). After excluding 93 aMCI participants, 27 aMCI–highEF and 26 aMCI‐lowEF participants were retained. Subsequently, 25 HCs were randomly included, with two individuals removed due to excessive head motion (cumulative translation or rotation > 3.0 mm or 3.0°), resulting in a final inclusion of 23 HCs. Ethical approval for the ADNI study was granted by the institutional review committees of all participating institutions. Participants or authorized representatives provided written informed consent.

### 2.2 Pathological sample acquisition

The cerebrospinal fluid levels of β‐amyloid protein (Aβ), total tau protein (t‐tau), phosphorylated tau protein (p‐tau), FDG‐PET data, and AV45‐PET data were obtained from the ADNI website (http://adni.loni.usc.edu). The details regarding processing methods were provided in Supporting Information.

### 2.3 MRI data acquisition

Detailed scanning information can be obtained from the ADNI website (http://adni.loni.usc.edu).

### 2.4 Functional data preprocessing

Preprocessing of fMRI data was performed using Data Processing and Analysis for Brain Imaging (DPABI, http://rfmri.org/DPABI) software in MATLAB 2019a (http://www.mathworks.com/products/matlab/). The preprocessing steps were provided in Supporting Information.

### 2.5 FC analysis

A seed‐based FC analysis was performed to explore the alternation of SMN. According to a previous study, 8 mm spherical regions of interest centered in the left hippocampus (montreal neurological institute space: −25, −15, −20) were created (Martersteck et al., 2020). Individual mean time series were extracted based on the coregistered seed region as the reference time series, and then a voxel‐wise cross‐correlation analysis was carried out between the seed region and the whole brain within the gray matter (GM) mask. Fisher's r‐to‐z transformation was used to improve the normality of the correlation coefficients.

### 2.6 Statistical analysis

Statistical Package for the Social Sciences (SPSS) software, version 22.0 (IBM), was employed for statistical analysis. The analysis of variance (ANOVA) and the chi‐square test were utilized to compare demographics, neurocognitive scales, and pathological index among the three groups: aMCI–highEF, aMCI‐lowEF, and HC. Bonferroni correction was applied for post hoc comparisons, and a *p* value of <.05 was considered statistically significant.

A one‐way ANOVA analysis was performed to compare the differences in FC of EMN after controlling for the influence of age, gender, and education level. Nonparametric permutation tests with threshold‐free cluster enhancement (TFCE) and family‐wise error (FWE) correction were employed for multiple comparisons. Clusters size > 50 voxels (1350 mm^3^) and *p*‐value < .05 after 1000 random permutations were considered statistically significant. Subsequently, a two‐sample *t*‐test was conducted using the brain regions that showed significant differences in covariance analysis, with significance defined as *p* < .05 (TFCE‐FWE correction) and cluster size > 50 voxels (1350 mm^3^).

Correlation analyses were performed in SPSS, investigating relationships between altered FC and cognitive function, adjusting for age, sex, and years of education as covariates (Bonferroni corrected, *p* < .05).

Receiver operating characteristic (ROC) curve analysis was carried out using SPSS 22.0 to assess the sensitivity and specificity of the altered FC in differentiating aMCI–highEF from aMCI‐lowEF.

## 3 RESULTS

### 3.1 Demographic and neurocognitive characteristics

Table 1 presents the data of demographic, neurocognitive, and pathological characteristics of all participants, including aMCI–highEF, aMCI‐lowEF and HC. Compared to HCs, aMCI–highEF exhibited reduced scores on the Mini‐Mental State Examination (MMSE) and immediate recall of the Rey Auditory Verbal Learning Test (RAVLT). Compared to HCs, aMCI‐lowEF had higher age and lower education levels. They also demonstrated significantly decreased scores on MMSE, Montreal Cognitive Assessment (MoCA), and immediate recall and learning scores of RAVLT, along with a significant increase in the RAVLT forgetting ratio. When comparing aMCI‐lowEF to aMCI–highEF, aMCI‐lowEF showed significantly lower education levels, MMSE scores, MoCA scores, immediate recall and learning scores of RAVLT, as well as FDG levels. Additionally, AV45 levels were significantly higher in the low executive function aMCI group (*p* < .05).

**TABLE 1:** Demographics and clinical and pathology data of three groups, including amnestic mild cognitive impairment (aMCI) with higher executive function (EF), aMCI with lower EF, and healthy control (HC).

	aMCI‐highEF (27)	aMCI‐lowEF (26)	HC (23)	*F*/*t* (*χ* ^2^)	*p* values
Age	69.652 ± 7.80	74.265 ± 6.89^*^	69.087 ± 5.63	4.322	.017^a^
Gender (male/female)	16/11	10/16	7/16	4.596	.100
Education level (years)	17.04 ± 2.26	14.85 ± 2.52^**^/^**^	16.91 ± 1.53	8.280	.001^ab^
MMSE	28.89 ± 1.19^***^	26.19 ± 2.30^***^/^***^	29.30 ± 1.15	26.747	<.001^abc^
MoCA	25.69 ± 1.67	19.60 ± 4.14^***^/^**^	27.04 ± 2.27	46.057	<.001^ac^
ADNI‐EF	1.916 ± 0.37	−0.839 ± 0.28^***^/^***^	1.172 ± 0.83	189.644	.001^ab^
RAVLT‐immediate	42.63 ± 5.54^**^	31.04 ± 10.87^***^/^***^	50.65 ± 10.43	28.468	<.001^abc^
RAVLT‐learning	5.81 ± 2.02	4.12 ± 2.78^*^/^*^	6.04 ± 2.60	4.595	.013^ab^
RAVLT‐forgetting	4.96 ± 2.82	3.92 ± 2.30	3.17 ± 3.26	2.588	.082
RAVLT‐percentage forgetting	45.17 ± 26.85	56.47 ± 33.75^**^	28.80 ± 31.35	4.979	.009^a^
Aβ	1042.46 ± 259.32	1027.83 ± 355.36		0.076	.941
t‐tau	262.53 ± 65.23	274.57 ± 91.36		0.277	.786
p‐tau	24.21 ± 5.83	26.05 ± 11.21		0.366	.720
FDG	1.30 ± 0.95	1.21 ± 0.15		2.220	.031
AV45	1.09 ± 0.15	1.27 ± 0.27		2.143	.043

*Note*: Numbers are given as means ± standard deviation, SD unless stated otherwise. Values for age derived from ANOVA; gender from chi‐square test; and all clinical measures from ANOVA and two‐sample *t* test.

Abbreviations: Aβ, β‐amyloid; aMCI‐highEF, amnestic mild cognitive impairment with higher executive function; aMCI‐lowEF, amnestic mild cognitive impairment with lower executive function; FDG, fluorodeoxyglucose; MMSE, Mini‐Mental State Examination; MoCA, Montreal Cognitive Assessment; p‐tau, phosphorylated tau protein; RAVLT, Rey Auditory Verbal Learning Test; t‐tau, total tau protein.

^a^Post hoc analyses showed a significantly group difference between aMCI–lowEF and HC

^b^Post hoc analyses showed a significantly group difference between aMCI–highEF and aMCI–lowEF.

^c^Post hoc analyses showed a significantly group difference between aMCI–highEF and HC.

^*^ *p* < .05; ^**^ *p* < .01; and ^***^ *p* < .001.

### 3.2 FC analysis

**FIGURE 1:**
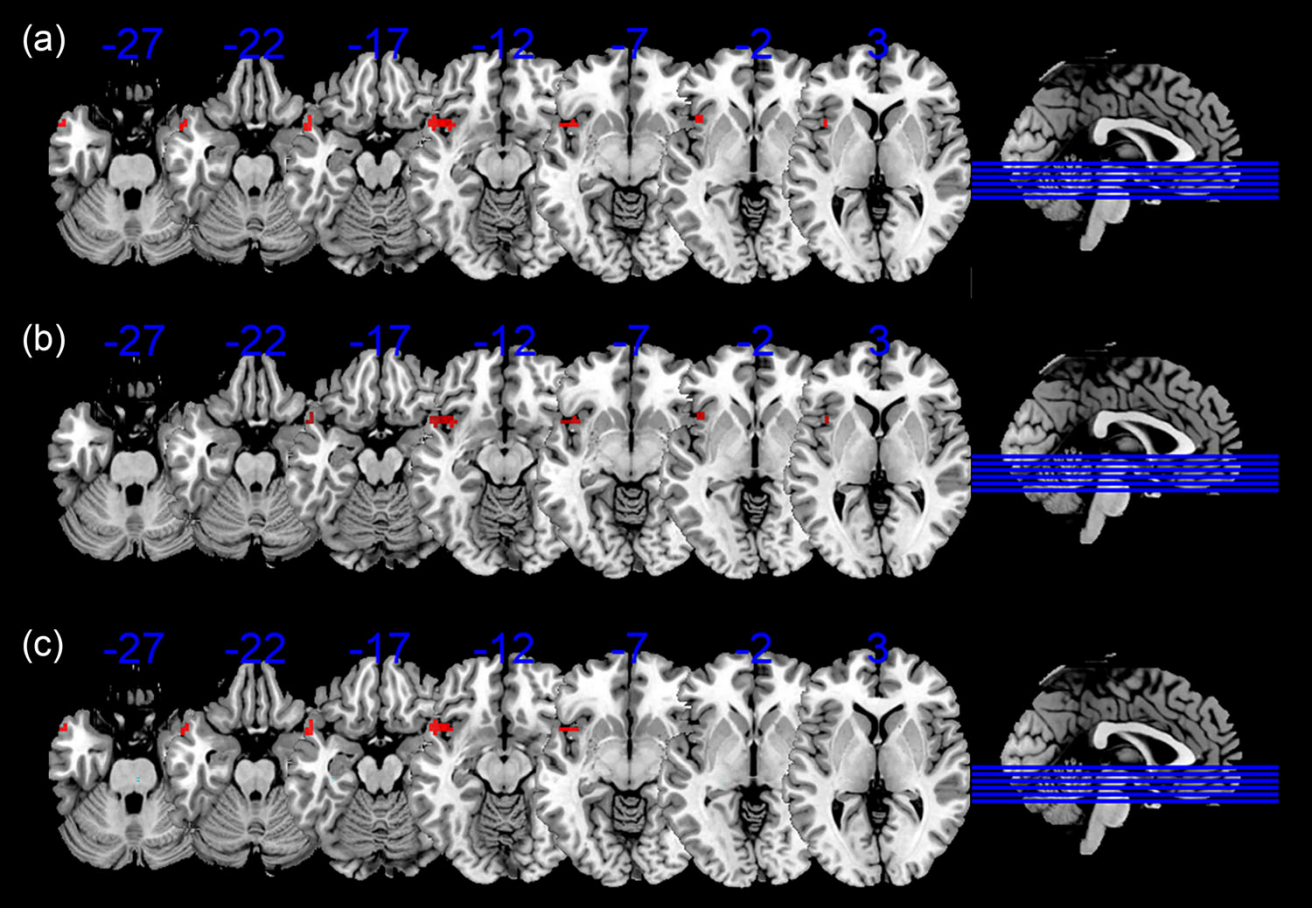
Brain regions exhibiting significant differences in functional connectivity in episodic memory network. (a) Brain regions showing significant differences in functional connectivity in the episodic memory network across three groups, including amnestic mild cognitive impairment with higher executive function (aMCI–highEF), amnestic mild cognitive impairment with lower executive function (aMCI–lowEF), and healthy control (HC) (threshold‐free cluster enhancement with family‐wise error [TFCE‐FWE] corrected, *p* < .05, the cluster size > 50 voxels). (b) Brain region showing significant differences in functional connectivity in the episodic memory network between aMCI–lowEF and HC (TFCE‐FWE corrected, cluster size > 50, *p* < .05). (c) Brain region showing significant differences in functional connectivity in the episodic memory network between aMCI–lowEF and aMCI–highEF (TFCE‐FWE corrected, cluster size > 50, *p* < .05).

The results of ANOVA among the three groups indicated a significant difference in FC in the left superior temporal gyrus (STG) after controlling for age, gender, and education level. Further pairwise comparisons revealed that, compared to both the HCs and aMCI–highEF group, the aMCI‐lowEF group exhibited increased FC in the left STG (TFCE‐FWE correction, *p* < .05, cluster size > 50 voxels) (Table 2; Figure 1).

**TABLE 2:** The difference of functional connectivity in episodic memory network across three groups.

Region	Peak MNI coordinate			*F*/*t*	Cluster number
	*x*	*y*	*z*		
ANOVA					
L superior temporal gyrus	−54	15	−15	8.903	78
aMCI‐lowEF versus HC					
L superior temporal gyrus	−39	12	−9	3.6231	58
aMCI‐lowEF versus aMCI‐highEF					
L superior temporal gyrus	−54	12	−24	3.7066	58

*Note*: The *x*, *y*, and *z* coordinates are the primary peak locations in the MNI space. Cluster size >19 voxels in ANOVA analysis, *p* < .05, TFCE‐FWE corrected. Cluster size >50 voxels in post hoc test, *p* < .05, TFCE‐FWE corrected.

Abbreviations: aMCI‐highEF, amnestic mild cognitive impairment with higher executive function; aMCI‐lowEF, amnestic mild cognitive impairment with lower executive function; MNI, montreal neurological institute; ANOVA, analysis of variance; HC, healthy control; L, left.

### 3.3 Correlation analysis

**FIGURE 2:**
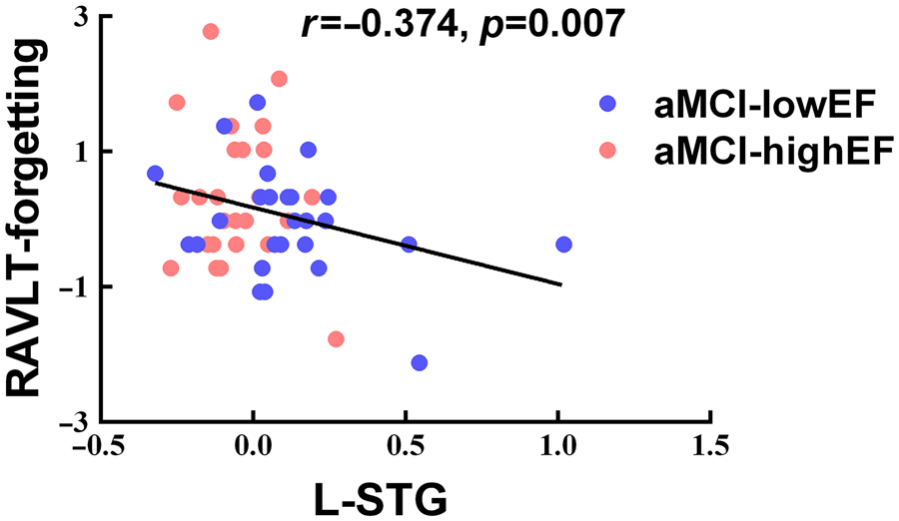
Brain regions exhibiting significant differences in functional connectivity in episodic memory network and the correlation with cognitive function. aMCI‐highEF, amnestic mild cognitive impairment with higher executive function; aMCI‐lowEF, amnestic mild cognitive impairment with lower executive function; L‐STG, left superior temporal gyrus; RAVLT, Rey Auditory Verbal Learning Test.

The correlation analysis indicated a significant negative correlation between the differences in FC in the left STG and the RAVLT forgetting score (*r* = −0.374, *p* = .007) (Figure 2).

### 3.4 ROC analysis

**FIGURE 3:**
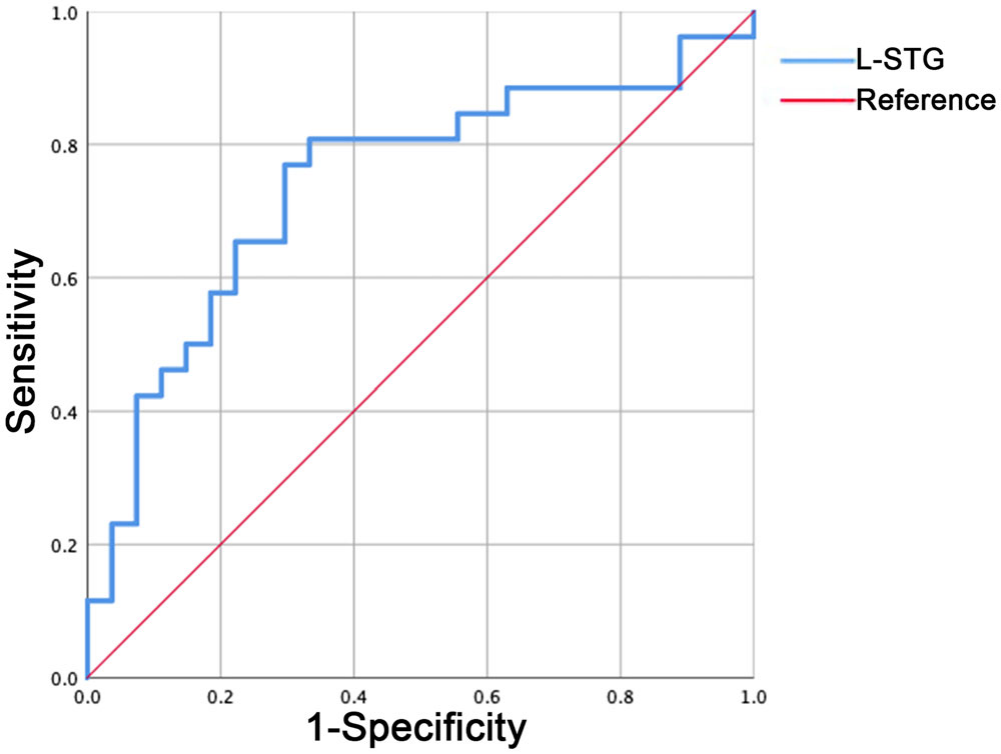
Diagnosis and differentiation of amnestic mild cognitive impairment with higher executive function (aMCI‐highEF) and amnestic mild cognitive impairment with lower executive function (aMCI‐lowEF) based on receiver operating characteristic (ROC) analysis. L‐STG, left superior temporal gyrus.

The area under the curve (AUC) for the differences in FC in the left STG in discriminating between the aMCI–highEF group and aMCI–lowEF group was 0.741, with a sensitivity of 0.808 and a specificity of 0.667 (Figure 3).

## 4 DISCUSSION

This study was the first to explore changes in the EMN based on different levels of EF. Several key findings have emerged from this research: first, aMCI patients with severe EF impairment exhibited more pronounced damage to the EMN, and this impairment was significantly correlated with EM. Second, alterations in FC in the left STG contributed to distinguishing aMCI patients with different levels of EF. This study validated the interaction between EF and EM, providing crucial insights for a deeper understanding of the pathological mechanisms underlying aMCI.

EF is consistently associated with EM performance in both childhood and old age (Dias et al., 2018). Previous researchers have proposed the “EF decline hypothesis,” suggesting that aging is not the core factor leading to memory decline; instead, EF also plays a crucial role (Lee et al., 2012; Salthouse et al., 2003). Studies suggest that the impact of aging on memory may result from the selective decline of specific EF (Ye et al., 2021). Individuals with aMCI also experience varying degrees of decline in EF, and impaired EF is correlated with a high progression rate from aMCI to AD (Aretouli et al., 2013; Xu et al., 2020). It is currently unclear whether the loss of EM and the decline in EF in aMCI patients are independent or interacting factors. The present study found that aMCI–lowEF group showed significant differences in MMSE, MoCA, and partial RAVLT scores compared to HC group/ aMCI‐highEF group. This indicated that the overall cognitive function and EM function were more severely impaired in the aMCI–lowEF group. This further confirmed that different levels of EF impact EM function in aMCI.

Previous research suggested that changes in cerebrospinal fluid Aβ precede the decline in EF before memory impairment. This aligned with the deposition of extracellular amyloid first appearing in the basal part of the same cortical areas before involving the hippocampus (Harrington et al., 2013). Consistent with previous studies, this research found an increase in AV45 in the aMCI–lowEF group compared to the aMCI‐highEF group. AV45 is a PET‐based measure of brain Aβ levels, and the decrease in AV45 validates a more pronounced Aβ deposition in aMCI with low EF (Tideman et al., 2022; Yoon et al., 2019). Furthermore, the current study found a decrease in FDG in the aMCI–lowEF group compared to the aMCI‐highEF group. This was also consistent with the fact that EF, combined with regional cerebral glucose metabolism, has high predictability for conversion from normal to MCI or AD dementia (Ewers et al., 2013). However, in this study, there were no significant differences in cerebrospinal fluid Aβ, t‐tau, and p‐tau proteins between the high and low EF aMCI groups, possibly due to the relatively small sample size.

In this study, there were no significant changes in the EMN of aMCI‐highEF suggesting that the integrity of EF may contribute to preserving the integrity of the EMN in aMCI. In contrast, aMCI–lowEF showed a significant increase in FC in the left STG compared to both aMCI–highEF and HCs. This further indicates that impaired EF affects the FC of the EMN. The STG is a crucial brain region in the default mode network, playing a key role in extracting meaningful language features and serving as a primary encoding center for temporal information (Jackson et al., 2018; L. Liu et al., 2023; Xue et al., 2019). Hänggi et al. (2011) found that the gray matter volume of the left STG has high sensitivity and specificity (92.3%/84.7%) in distinguishing between AD and aMCI.

The correlational analysis in this study revealed a significant negative correlation between the FC of the left STG and the forgetting score of the RAVLT. As EM declined, the FC of the left STG increased, suggesting a potential compensatory mechanism. The result proved that the left STG is a shared substrate for auditory short‐term memory and speech comprehension (Leff et al., 2009). Yue et al. also found alterations in the amplitude of low frequency fluctuation in the STG of aMCI patients, positively correlated with MoCA scores, proposing that abnormalities in the STG might be related to dysfunction across multiple cognitive domains, consistent with the findings of this study (Yue et al., 2023). Furthermore, ROC analysis demonstrated that the FC of the left STG contributes to distinguishing aMCI with different levels of EF. This further confirms the interaction between EF and EM. Therefore, the left STG may serve as a significant biological marker for aMCI, offering new insights into targeting for early intervention. In summary, this study explored the differences in EMN at various levels of pathology, cognition, and imaging. It was confirmed that EF mediates the EMN in individuals with aMCI, thereby impacting the EM in aMCI. This finding holds significant implications for enhancing our understanding of the pathological mechanisms underlying AD conversion, clinical prediction of AD conversion, and early intervention strategies.

### 4.1 Limitations

This study had some limitations. First, the data for this research were derived from the ADNI database, primarily representing European and American populations. Due to differences in genetic backgrounds, lifestyles, and environmental factors, the generalizability of the results to other regions may be limited. Therefore, further validation was needed to determine the applicability of the findings to different races with AD. Second, the sample size in this study was relatively small, potentially affecting statistical power, increasing the risk of type II errors, and reducing generalizability. To mitigate potential sample size effects, rigorous correction methods were employed in the present study. Additionally, longitudinal studies will be conducted in the future to track and follow up on individuals with aMCI, allowing for a more comprehensive examination of the evolution of EF in aMCI patients transitioning to AD. This will enhance our understanding of the role EF plays in AD progression.

## 5 CONCLUSIONS

In summary, this study confirmed characteristic changes in the FC of the EMN in aMCI with impaired EF, which was significantly correlated with EM. This provided theoretical support for the notion that EF mediates the EMN, thereby influencing EM. Additionally, the study revealed a more pronounced deposition of pathological markers in individuals with impaired EF, suggesting that the level of EF may serve as an effective biomarker for predicting the progression of AD. In conclusion, a multimodal approach integrating EF levels, EMN changes, and pathological features holds significant value in predicting the outcomes of aMCI.

## AUTHOR CONTRIBUTIONS

Chao Wang, Rukun Cheng, Lin Qiu, and Haifeng Liu designed the study. Chao Wang, Rukun Cheng, Wenhao Yang, Lin Qiu, and Haifeng Liu collected the data. Chao Wang, Rukun Cheng, Lin Qiu, and Haifeng Liu analyzed the data and prepared the manuscript.

## CONFLICT OF INTEREST STATEMENT

The authors declare no conflicts of interest.

### PEER REVIEW

The peer review history for this article is available at https://publons.com/publon/10.1002/brb3.3601.

## Supporting information

Supporting Information

## Data Availability

Data used in preparation of this article were obtained from the Alzheimer's Disease Neuroimaging Initiative (ADNI) database (adni.loni.usc.edu).
