# Supplementary material for: Episodic memory network characteristics in patients with amnestic mild cognitive impairment accompanied by executive function impairment
Source: Brain Behav. 2024 Jun 19;14(6):e3601. doi: 10.1002/brb3.3601 (PMC11186851; doi:10.1002/brb3.3601)
Supplement: Supplementary file 1 — Supporting Information [file BRB3-14-e3601-s001.docx]

***SI Methods***

**Pathological sample acquisition**

The INNO-BIAALZBio3 immunoassay kit was used to determine cerebrospinal fluid levels of β-amyloid protein (Aβ), total tau protein (t-tau), and phosphorylated tau protein (p-tau).

FDG-PET data were based on the Automated Anatomical Labeling (AAL) template, extracting standardized values of fluorodeoxyglucose (FDG) uptake in five AD-sensitive target regions, including the right and left middle/inferior temporal lobes, right and left angular gyri, and bilateral posterior cingulate gyri relative to the pons/medulla. FDG levels reflect brain glucose metabolism.

AV45-PET data involved averaging AV45 uptake values in bilateral frontal lobes, lateral parietal lobes, lateral temporal lobes, anterior/posterior cingulate gyri, and left and right hippocampal regions. These values were standardized to a composite reference region (including the entire cerebellum, brainstem/pons, and subcortical white matter). AV45-PET is capable of detecting brain Aβ levels. All participant pathology information and processing methods were obtained from the ADNI website ([http://adni.loni.usc.edu](http://adni.loni.usc.edu/)).

**Functional data preprocessing**

Preprocessing of fMRI data was performed using Data Processing and Analysis for Brain Imaging (DPABI, http://rfmri.org/DPABI) software in MATLAB 2019a (http://www.mathworks.com/products/matlab/). The preprocessing steps were included: 1) removal of the first 10 time points for each participant; 2) slice timing correction; 3) head motion correction: exclusion of participants with horizontal displacement >3.0 mm or rotational motion >3.0°; 4) spatial normalization: each participant was spatially normalized to the Montreal Neurological Institute (MNI) echo-planar imaging template and resampled to 3 × 3 × 3 mm^3^ voxels; 5) regression of covariates to remove nuisance variables such as white matter, cerebrospinal fluid, and whole-brain signal; 6) smoothing using a 6 mm full-width at half-maximum Gaussian kernel; 7) low-frequency filtering (0.01 ~ 0.08 Hz).
